# Towards a secure Metaverse: Leveraging hybrid model for IoT anomaly detection

**DOI:** 10.1371/journal.pone.0321224

**Published:** 2025-04-14

**Authors:** Sanchit Vashisht, Shalli Rani, Mohammad Shabaz

**Affiliations:** 1 Chitkara University Institute of Engineering and Technology, Chitkara University, Punjab, India; 2 Model Institute of Engineering and Technology, Jammu, Jammu and Kashmir, India; Gachon University, KOREA, REPUBLIC OF

## Abstract

The seamless interaction between the virtual and real worlds is due to the unprecedented degrees of decentralization, immersiveness and connectedness made possible by the Internet of Things (IoT) and the metaverse. In this light, it brings important ethical, privacy, and security considerations into play, hence calling for the strong protection of IoT-enabled metaverse systems. Anomaly detection is critical for solving the aforementioned issues and ensuring the dependability and security of the connected devices by identification and preventing malicious activity in IoT networks. With IoT networks being highly dynamic and complex, robust anomaly detection frameworks are essential for ensuring security and trust in the metaverse. This paper proposed a hybrid model combining Random Forest (RF) and Neural Network (NN) and compared it with a variety of machine learning (ML) techniques including Decision Tree (DT), Naive Bayes (NB), K-Nearest Neighbor (KNN), RF and Logistic Regression (LR) to detect anomalies in IoT-enabled metaverse environments. These models were trained and tested using the CIC-IDS 2017 Network Intrusion Dataset, a comprehensive benchmark used for evaluating intrusion detection systems (IDS). Indeed, with outstanding accuracy equaling a staggering 99.99%, the proposed hybrid model algorithm performed better than other ML models under study. This illustrates its vast potential for high-accuracy anomaly identification and false positives.

## 1 Introduction

The Metaverse unites internet technologies, such as virtual reality (VR) and augmented reality (AR), to bring about an all-inclusive digital experience [1]. This field has the potential to change industries, such as entertainment, education, and healthcare, because it links the virtual world with the physical world and offers an enhanced user experience that requires real-time data and interactive features [2]. Determining who owns the data produced by IoT devices in the Metaverse is a major difficulty. Consent concerns, illegal data access, and possible misuse emerge from the continuous transmission of sensitive user information [3]. Self-sovereign identity (SSI) and decentralized identity management (DID) approaches can be used to increase trust in IoT-enabled Metaverse systems. By guaranteeing transparent and unchangeable record-keeping and giving users control over who can access their personal data, blockchain technology can significantly strengthen data ownership rights. Furthermore, ML models can function on decentralized data without disclosing sensitive information because to privacy-preserving strategies like federated learning and differential privacy. The Metaverse can create a more safe and user-trusted environment by resolving these ethical issues, guaranteeing fair access while upholding strong security protocols [4].

**Fig 1:**
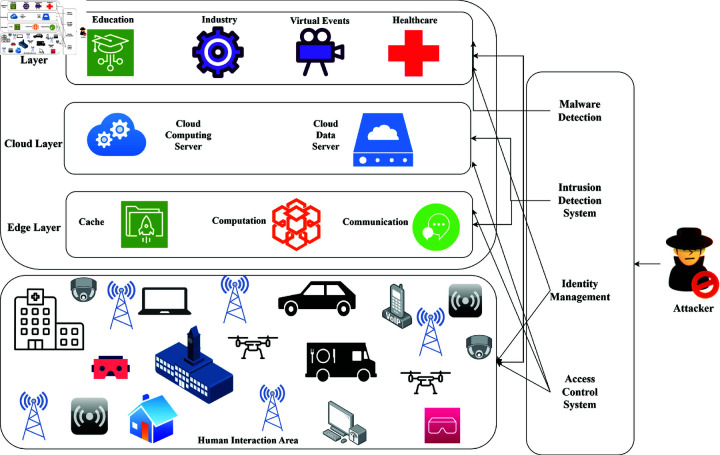
Architecture of IoT-enabled metaverse.

For this reason, IoT is vital for this change. IoT devices that support personal virtual worlds are wearables, smart sensors, and actuators [5]. For example, wearable technology uses fitness data to create personalized activities in virtual environments, and smart home devices enhance interactions by fusing digital avatars with environmental sensors [6]. Consumer devices are the backbone of IoT applications in the Metaverse [7]. Daily appliances include fitness trackers, smartwatches, cellphones, and home automation systems connecting users to virtual worlds [8]. These devices make possible features like motion detection, biometric tracking, and communication in order to achieve realistic and immersive Metaverse experiences [9,10]. Due to mass usage and interconnectivity, however, these bring forth significant cybersecurity challenges. A compromised device in a Metaverse network can be used as a stepping stone for further attacks that compromise user privacy, disrupt services, and break trust [11]. Fig 1 represents a three-tier architecture of a secure Metaverse ecosystem. The Application Layer shows some important sectors of the Metaverse: healthcare, education, industry, and virtual events [12]. The Cloud Layer is a cloud computing and data servers, providing centralized data processing. The parts of the Edge Layer include communication, computation, and caching to enable real-time interactions in human interaction scenarios. Other measures include identity management, virus detection, intrusion detection, and access control systems for protecting against data breach and unauthorized user information access [13].

Anomaly detection is vital to minimizing the threats associated with applications of IoT within the Metaverse [14]. Advanced systems for detection identify unusual network traffic or patterns in device activities and prevent all the above forms of threats: DDoS attacks, unauthorized access, and data breaches. The lack of such anomaly detection systems gravely threatens user trust, data privacy, and integrity in virtual settings as well [15]. The problem is further complicated by the complexity of IoT networks, with a large variety of devices, communication protocols, and data types. Thus, complex and scalable solutions are required [16]. Additionally, the problem of privacy and ethics is equally crucial for IoT-enabled Metaverse applications. Concerning the important contextual and personal data that are constantly being transmitted from consumer devices to the Metaverse platform, issues arise about the ownership of the data, consent, and possibly illegal monitoring or profiling [17]. In system design, it is crucial to protect users by focusing on user-centered privacy safeguards and ethical standards and by maintaining strong security protocols in order to ensure equal access to the Metaverse. This will address these problems and enhance consumer trust to unlock the transformative potential of the Metaverse [18,19]. ML techniques are revolutionizing anomaly detection in IoT-enabled Metaverse scenarios [20]. Among the most effective ML algorithms used for anomaly detection in large and complex datasets, RF, DT, NB, KNN, and LR that can also give real-time alarms on potential risks [21]. These technologies have been quite effective in dealing with security issues due to their high capability of enhancing accuracy in detection, false alarms reduction, and response to new attacks [16,22].

### 1.1 Objectives

- Evaluate and compare the effectiveness of different ML models to the proposed model to detect irregularities in IoT-enabled metaverse using the CIC-IDS dataset.
- Address the security vulnerabilities of IoT-enabled Metaverse applications and preserve user privacy with state-of-the-art comparison.
- Proposed a hybrid model for anomaly detection that can be utilized in the complex and dynamic virtual IoT-enabled metaverse environments.

## 2 Relatedwork

One of the most attention-grabbing recent topics of discussion is the integration of IoT into Metaverse scenarios, with the focus lying more on security and privacy issues. Studies consider various anomaly detection techniques, which range from ML to DL models, to find and solve issues related to IoT networks. Such techniques as hybrid frameworks and unsupervised learning are promising tools in detecting novel, previously unknown threats in dynamic IoT networks. Even with the current progression, designing adaptable and scalable solutions which are in line with moral standards as well as provide sound security for different applications remains a tough nut to crack. This section discusses the landmark contributions and the gaps motivating the present study.

### 2.0.1 Based on machine learning

Sarker et al. [23], presented “IntruDTree” model, a machine-learning-based intrusion detection system that prioritizes security features and develops a tree-based model to enhance prediction accuracy while reducing computational complexity. The model outperforms traditional classifiers such as NB, LR, SVM, and KNN in terms of recall, precision, F-score, and ROC metrics. The limitation lies in its dependence on the quality of feature ranking and dataset representation, which may affect its generalizability to diverse cyber-attack scenarios. Liu et al. [24], detected of IoT-specific attacks using the NSL-KDD dataset and evaluates eleven ML algorithms. Tree-based and ensemble methods, particularly XGBoost, achieved the highest performance, with 97% accuracy, 90.5% MCC, and 99.6% AUC. The Expectation-Maximization (EM) algorithm, an unsupervised method, demonstrated a 22% improvement in accuracy over NB. The dataset’s homogeneity poses a significant limitation, potentially compromising its capacity to accurately represent real-world IoT environments and restricting generalization potential.

Yu et al. [25] designed an intrusion detection system that has applied FSL to address anomalous samples shortage in network behavior datasets. The approach attained a classification accuracy of 92.34% on KDD-Test+ and 85.75% on KDD-Test-21 with the training data usage less than 1% of the size of the NSL-KDD dataset and better performance compared with traditional methods including J48, NB, and RF. However, the structure dependency on the dataset limits scalability and adaptability to other different network environments. Gao et al. [26] proposed an adaptive ensemble learning scheme to solve the problems with the traditional intrusion detection schemes. This was a development based on the NSL-KDD dataset. The MultiTree algorithm and adaptive voting ensemble were able to obtain 84.2% and 85.2% accuracies, respectively, which outperformed standalone models, including DT and kNN. The research underscored the importance of data quality in ensuring accurate identification. Limitations involve dependence on specific features of NSL-KDD and the better feature selection/preprocessing for a better generalization across different datasets.

Gu et al. [27] introduced an intrusion detection framework that combines SVM with NB feature embedding to enhance data quality. The NB transformation improves feature quality and enhances SVM classification performance. The framework exhibits robust performance, attaining accuracies of 93.75% for UNSW-NB15, 98.92% for CICIDS2017, 99.35% for NSL-KDD, and 98.58% for Kyoto 2006+. The limitation lies in its reliance on computationally intensive transformations, which may impede scalability for real-time intrusion detection in high-traffic networks. Kurniawan et al. [28] enhanced the NB algorithm to address prediction issues associated with zero probabilities. Two modifications are implemented: the removal of variables with zero probabilities and the replacement of multiplication with addition operations. The adjustments improve the algorithm’s precision, recall, and accuracy by up to 4%, 2%, and 2%, respectively, when compared to the original method. However, a limitation remains as the modifications may not address other inherent issues of NB, such as the handling of correlated features.

### 2.0.2 Based on deep learning

Murgai et al. [29] explored a new method to discover and categorize apps in order to strengthen the security of the Metaverse with an open-source dataset. The applications were classified into three groups: network infrastructure, real-time conversational, and non-real-time. Anomaly detection results of 85% with XGBoost and 87% with DNN models. Application behaviors related to zero-byte packets were found to be "unclassified" by the anomaly detection model, which might indicate port-snooping or DoS attacks. The study has limitations in the vastness of the collection and the difficulty of identifying rare anomalies. Gupta et al. [30] proposed a CNN-based DL model to detect unusual patterns in centralized Metaverse systems. The model reached an accuracy of 94.73% and a test loss of 0.206631 after ten training cycles. In high-dimensional data, the model excels LR and Feedforward Neural Networks in terms of pattern recognition. Despite the success of the paper, the lack of diversity in the data may limit the method’s applicability to more extensive real-world Metaverse settings.

Rathore et al. [31] proposed a threat detection framework for fog-based systems. For the prevention of security threats in the IoT, they applied ELM-based Semi-supervised Fuzzy C-Means. Distributed attack detection is done along with the removal of labeled data problems using fog computing to improve scalability and reduce latency. Testing on the NSL-KDD dataset produced an accuracy of 86.53% and a detection time of 11 ms. Limitations include reliance on the specific dataset and possible scalability problems across other IoT configurations. Musleh et al. [32], analyzed ML-based IDS within IoT environments, emphasizing the importance of feature extraction techniques like VGG-16 and DenseNet. Various ML algorithms, including RF, KNN, and stacked models, were evaluated using the IEEE Dataport dataset. In a stacking approach with VGG-16, it achieved an accuracy of 98.3%. The disadvantages include reliance on computationally demanding feature extraction models and the need for optimization over specific datasets that may not be easily generalizable across different IoT environments. The comparison of existing methodologies as studies from literature were carried out in Table 1.

**Table 1:** Comparison of existing approaches.

Study	Dataset	Method	Accuracy	Limitations
Sarker et al. [23]	NSL-KDD	Decision Tree	93%	Lacks adaptability to evolving attacks
Liu et al. [33]	NSL-KDD	XGBoost	97%	Dataset lacks real-world diversity
Gupta et al. [34]	Custom	CNN	94.73%	High computational cost
Gao et al. [26]	NSL-KDD	Adaptive Ensemble	85.2%	Overfitting on dataset

#### 3 Dataset and methodology

##### 3.1 Dataset

The CIC-IDS 2017 Network Intrusion Dataset, a popular dataset for network intrusion research and anomaly detection, is used in this study is available at https://www.kaggle.com/datasets/chethuhn/network-intrusion-dataset. This dataset captures actual network traffic. Routine operations as well as several types of cyberattacks, including brute-force, denial-of-service (DoS), and infiltration efforts, are included in this traffic. The dataset’s wide range of features, including labeled records, flow-level statistics, and packet-level data, make it suitable for both training and testing of ML models. All the features in this dataset are shown in Table 2.

**Table 2:** Features of the CIC-IDS 2017 network intrusion dataset.

Features	Features	Features
Destination.Port	Flow.Duration	Total.Fwd.Packets
TotalBackward Packets	TotalLength of FwdPackets	TotalLength of BwdPackets
FwdPacket LengthMax	FwdPacket LengthMin	FwdPacket LengthMean
FwdPacket LengthStd	BwdPacket LengthMax	BwdPacket LengthMin
BwdPacket LengthMean	BwdPacket LengthStd	FlowBytes/s
FlowPackets/s	FlowIAT Mean	FlowIAT Std
FlowIAT Max	FlowIAT Min	FwdIAT Total
FwdIAT Mean	FwdIAT Std	FwdIAT Max
FwdIAT Min	BwdIAT Total	BwdIAT Mean
BwdIAT Std	BwdIATM in	BwdIAT Max
Fwd PSHFlags	Bwd PSHFlags	Fwd URGFlags
Bwd URGFlags	FwdHeader Length	BwdHeader Length
FwdPackets	BwdPackets	MinPacket Length
MaxPacket Length	PacketLength Mean	PacketLength Std
PacketLength Variance	FINFlag Count	SYNFlag Count
RSTFlag Count	PSHFlag Count	ACKFlag Count
URGFlag Count	CWEFlag Count	ECEFlag Count
Down/Up Ratio	AveragePacket Size	AvgFwdSegment Size
AvgBwd SegmentSize	FwdHeader Length.1	FwdAvgBytes Bulk
FwdAvgPackets Bulk	FwdAvgBulk Rate	BwdAvgBytes Bulk
BwdAvgPackets Bulk	BwdAvgBulk Rate	SubflowFwd Packets
SubflowFwd Bytes	SubflowBwd Packets	SubflowBwd Bytes
InitWinbytes forward	InitWinbytes backward	actdatapkt fwd
minsegsize forward	ActiveMean	ActiveStd
ActiveMax	ActiveMin	IdleMean
IdleStd	IdleMax	IdleMin
Label		

#### 3.2 Methodology

**Fig 2:**
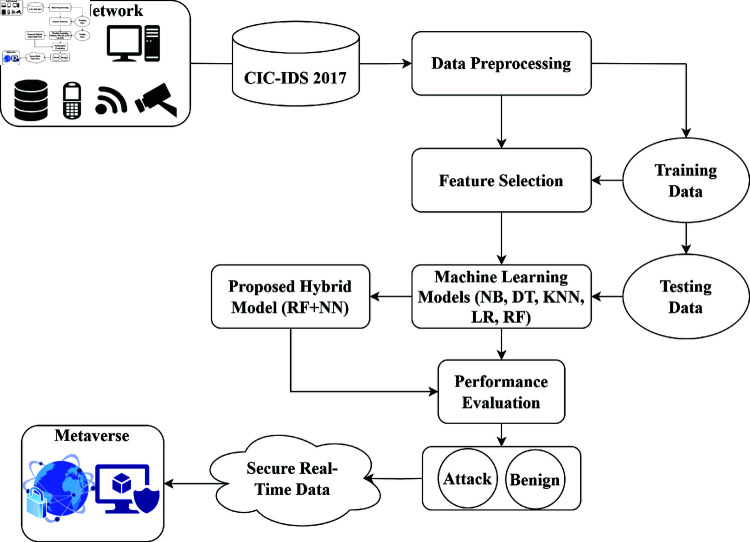
Proposed methodology.

This section outlines the systematic approach taken in this study, which comprised feature selection, data pre-processing, and the application of a ML model. By employing a systematic approach, the research aimed to optimize the models’ prediction performance and reliability. Fig 2 illustrates a security workflow for IoT-based metaverse systems. An IoT Network generates data, which is processed with the CIC-IDS 2017 dataset for ID. The workflow includes data preprocessing, feature extraction, and the division of data into training and testing subsets. A proposed hybrid Model classifies data into attack or benign categories well, with performance results evaluated accordingly. This process guarantees secure real-time data flow in the metaverse, facilitating dependable user interactions.

##### 3.2.1 Data preprocessing

The most crucial step in making sure that the dataset used is normalized, standardized and is ready for implementation for the model training and testing is termed as data preprocessing. Firstly erroneous, duplicate, or missing entries are rectified in the raw dataset. The appropriate imputation approaches were used to address missing values, such as mode replacement for categorical variables and mean or median replacement for numerical data [35]. Outliers were identified and addressed using statistical techniques such as z-scores, and duplicate entries were eliminated to prevent repetition. In order to make all categorical variables compatible with ML approaches, label encoding was also used. Ultimately, the dataset was standardized or normalized to help scale-sensitive algorithms by bringing all attributes to a same scale. In a 67:33 split of the dataset, 67% was used for training and 33% for model testing [36].

##### 3.2.2 Feature selection

**Fig 3:**
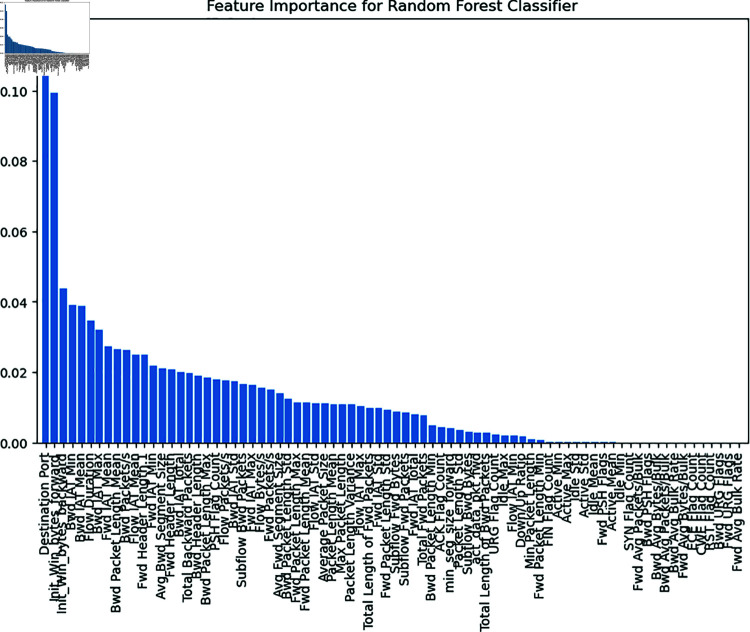
Selected features using the random forest classifier.

Feature selection boost performance of model by detecting the most crucial variables and reducing their computational complexity. RF was employed for this because it can rank features based on how important they are for making decisions. The algorithm calculates feature significance scores by looking at the decrease in node impurity that happens when a feature is used for splitting. While low-scoring features were eliminated, high-scoring features were retained in the final dataset. This decision minimized noise and improved the interpretability of the results. For this study, RF is used to rank features by importance, retaining only the most influential ones. Eliminating low-scoring features reduces model complexity and computational overhead. These features were then applied to all ML models. Fig. 3 illustrates the important features obtained from an RF Classifier for ID in a metaverse system. The x-axis displays the individual features in the dataset, including “Destination Port,” “Flow Duration,” and “Bwd Packet Length,” whereas the y-axis reflects their relative importance. Features such as “Destination Port” and “Flow Duration” exhibit the greatest predictive significance, as evidenced by their larger bars. This analysis identifies the key factors for accurately classifying network traffic as either attack or benign.

##### 3.2.3 Machine learning models

A variety of ML models were employed to compare and identify the effective solution for the issue at hand. These models include:

- Naive Bayes: A probabilistic classifier based on the Bayes principle and predicated on feature independence. It is computationally efficient and performs well with datasets that contain categorical variables. Eq 1 shows the mathematical equation of NB.
  $$\begin{array}{ccc} P(z|X)=\frac{P(X|z)P(z)}{P(X)} & & \end{array}$$ (1)
  where *P* ( *z* | *X* ) , *P* ( *X* | *z* ) , *P*(*z*) and *P*(*X*) are the posterior probability of class *z* given input *X*, likelihood of observing *X* given class *z*, prior probability of class *z* and the marginal probability of input *X* across all classes respectively.
- Decision Tree: A tree-structured method that separates data based on feature thresholds to produce a hierarchy of options. It is easy to use and does a good job at handling non-linear interactions. Eq 2 depicts the mathematical representation of impurity measure like Information Gain to split the data.
  $$\begin{array}{ccc} Gini=1-\overset{n}{\underset{i=1}{\sum }}P_{i}^{2} & & \end{array}$$ (2)
  where $P_{i}$ and *n* are the probability of class *i* in a given node and total number of classes respectively.
- K-Nearest Neighbor: A non-parametric method for classifying instances based on the majority class of the k-nearest data points. It uses distance measures and scaled data. Eq 3 represents the distance between points and neighbors using the Euclidean Distance.
  $$\begin{array}{ccc} d(w_{i},w_{j})=\sqrt{\overset{n}{\underset{k=1}{\sum }}{\left(w_{i,k}-w_{j,k}\right)}^{2}} & & \end{array}$$ (3)
  where $w_{i}∕w_{j}$, *k* and *n* are the feature vectors of two data points, feature index and the total number of features respectively.
- Logistic Regression: A linear model for binary classification that determines the probability that an instance is a member of a specific class. LR is an effective and easy to understand model, it works well with linearly separable data. Eq 4 shows the probability of a binary outcome utilizing the sigmoid function.
  $$\begin{array}{ccc} P(z=1|X)=\frac{1}{1+e^{-z}} & & \end{array}$$ (4)
  where *P* ( *z* = 1 | *X* ) , *e* and *z* are the probability of class *z* = 1 given input *X*, Euler’s number and Linear combination of input features respectively.
- Random Forest: Stacking many DTs enhances resilience and accuracy in this ensemble learning approach. It is quite useful for feature-rich datasets since it allows one to regulate high-dimensional spaces and avoid overfitting. Equipped with several decision trees, Eq 5 compiles predictions for majority-based final result projection.
  $$\begin{array}{ccc} ŷ=\frac{1}{m}\overset{m}{\underset{i=1}{\sum }}y_{i} & & \end{array}$$ (5)
  where *ŷ*, $y_{i}$ and *m* are the final predicted value, individual prediction from the *i*-th DT and the total number of DTs respectively.
- Neural Network: An NN is a mathematical model that takes idea from real brain networks’ architecture and behavior. It is composed of layers of interconnected nodes, or neurons, with a weight assigned to each connection. Because they can identify patterns in data, neural networks are frequently employed for tasks including feature extraction, regression, and classification. The forward propagating step in a NN’s one-layer structure is represented by Eq 6.
  $$\begin{array}{ccc} z=W\cdot X+b;a=\sigma (z) & & \end{array}$$ (6)
  where *W*, *X*, *b*, *z* and *σ* ( *z* )  are the weight matrix of the neural network layer, input feature vector, bias term, linear transformation before activation and Activation function (e.g., ReLU, Sigmoid), which introduces non-linearity respectively.

## 4 Results and analysis

This section discusses the implications for applications in the IoT-enabled Metaverse and demonstrates the results of applying ML methods to identify anomalies in the CIC-IDS 2017 Dataset. The results provide a detailed comparison of the effectiveness of various models in IDS by evaluating the performance of the models in terms of precision, recall, accuracy, and F1-score. Special emphasis is provided to the identification capabilities of models in various forms of attacks, due to their immense importance in protection of IoT ecosystems in the Metaverse. An investigation is given on how they improve the architecture of security that Metaverse applications make use of as well as its flexibility in dynamically changing, multiple device IoT systems. These findings inform the development of trustworthy, scalable anomaly detection systems that respect ethical and privacy standards.

### 4.1 Confusion matrix

A confusion matrix is the performance evaluation of statistical classification or machine learning, representing a summary of the prediction result of a classification model. The matrix contrasts expected labels with the actual labels; this square matrix can be applied to compute essential performance metrics like precision, accuracy, recall, and F1-score, all of which bring more details about how well a model performs and perhaps where it has scope for improvement.

- True Positives (TP): Instances that are correctly classified where the actual class is positive.
- True Negatives (TN): Instances that are correctly classified where the actual class is negative.
- False Positives (FP): Instances that are incorrectly classified as positive when the actual class is negative.
- False Negatives (FN): Instances that are incorrectly classified as negative when the actual class is positive.

**Fig 4:**
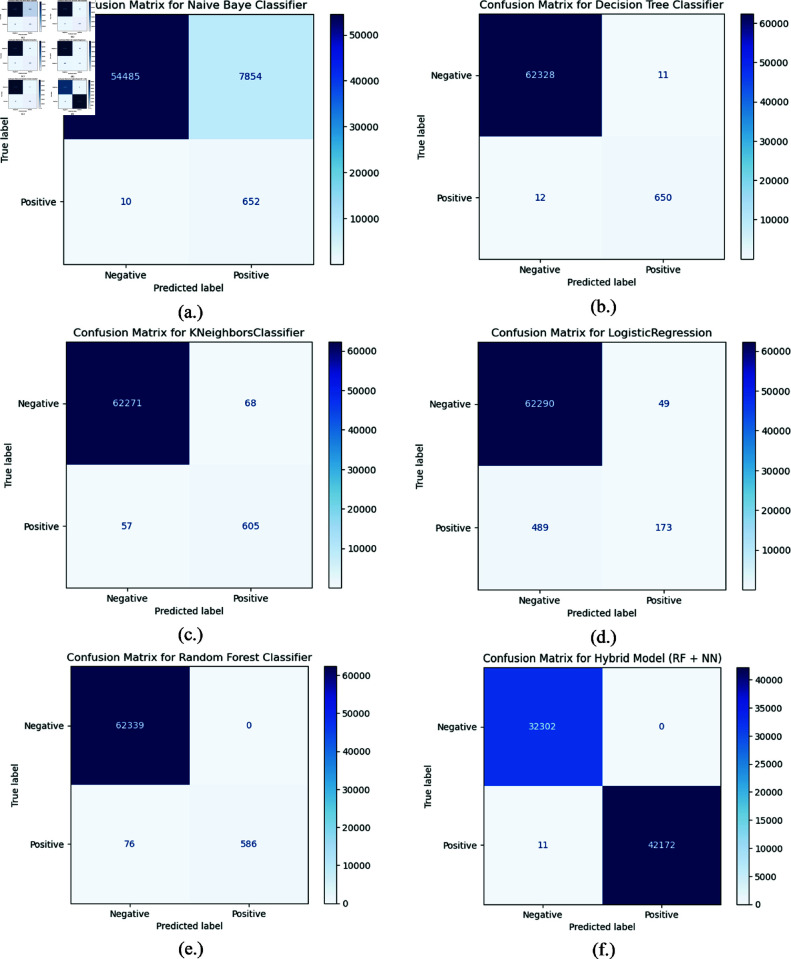
Confusion matrices of (a) NB, (b) DT, (c) KNN, (d) LR, (e) RF, (f) proposed hybrid.

The confusion matrices of NB, DT, KNN, LR, RF and proposed hybrid model are represented by the Fig 4 respectively. The figure displays the TP, FP, TN, and FN values for each algorithm. Whereas, Table 3 shows the hyperparameter settings for the different ML models to reduce the false positive rate for better accuracy. Different hyperparameter combinations were examined to assess model performance. Hyperparameters like 100 trees and max depth of 12 for RF and 3-layer architecture with activation of ReLU for NN consistently yielded the best accuracy, precision, and recall. Other settings caused overfitting, computational complexity, or poor detection. Thus, the selected hyperparameters balance accuracy and efficiency, making them ideal for identifying anomalies in IoT-enabled metaverse contexts.

**Table 3:** Hyperparameter settings for ML models.

Model	Hyperparameter	Value
NB	Smoothing Parameter (*α*)	1.0
	Binarize	0.0
DT	Criterion	Entropy
	Max Depth	10
	Min Samples Split	2
KNN	Number of Neighbors (*k*)	5
	Metric	Minkowski
	Weights	Uniform
LR	Solver	lbfgs
	Regularization (C)	1.0
	Maximum Iterations	100
RF	Number of Trees (n_estimators)	100
	Criterion	Entropy
	Max Depth	12
NN	Number of layers	3
	Hidden layer sizes	(50, 25)
	Maximum iterations	300
	Activation function	ReLU, sigmoid
	Optimized	Adam

### 4.2 Accuracy

Accuracy measures the proportion of correctly classified instances among the total instances. It is mathematically calculated as Eq 7.


$$\begin{array}{ccc} \text{Accuracy}=\frac{\text{TN}+\text{TP}}{\text{TP}+\text{FP}+\text{TN}+\text{FN}} & & \end{array}$$ (7)

**Fig 5:**
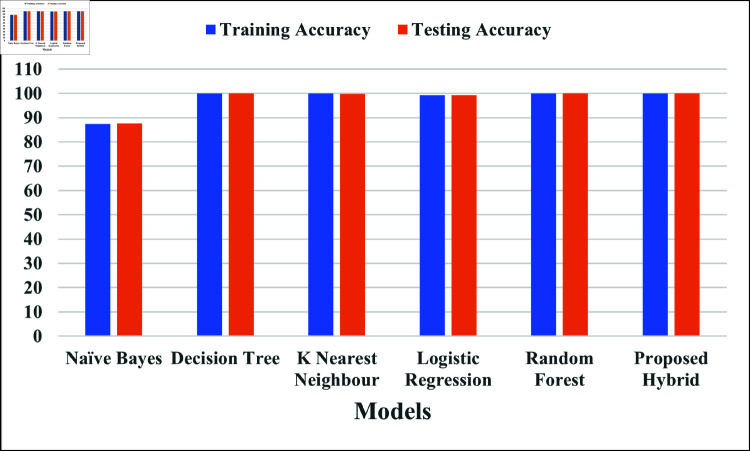
Comparison of proposed hybrid model with different ML model with regards to training and testing.

Fig. 5 illustrates the learning performance and generalization capacity of the models by contrasting their training and testing accuracies. The training accuracies of NB, DT, KNN, LR, RF and proposed hybrid model are 87.45%, 100%, 99.86%, 99.16%, 99.92% and 99.98% respectively, and the testing accuracies are NB, DT, KNN, LR, RF and proposed hybrid model are 87.51%, 99.96%, 99.8%, 99.14%, 99.87% and 99.99% respectively. While comparable high accuracy values of hybrid model imply robust performance, large differences between training and testing accuracy point to overfitting.

Table 4 shows the comparison of proposed hybrid model With other ML models in terms of performance metrics including accuracy, precision, recall and F1-score.

**Table 4:** Comparison of proposed hybrid model with other ML models in terms of performance metrics.

	Performance metrics(%)				
Models	Training accuracy	Testing accuracy	Precision	Recall	F1-score
NB	87.45	87.51	7.67	98	14.22
DT	100	99.96	98	98	98
KNN	99.86	99.8	89	91	39
LR	99.16	99.14	77	26	93
RF	99.92	99.87	100	88	93
Proposed Hybrid	99.98	99.99	100	99.97	99.98

### 4.3 Precision

Precision measures the proportion of correctly anticipated positive instances out of all anticipated positive instances. It is mathematically calculated as Eq 8.


$$\begin{array}{ccc} \text{Precision}=\frac{\text{TP}}{\text{TP}+\text{FP}} & & \end{array}$$ (8)

**Fig 6:**
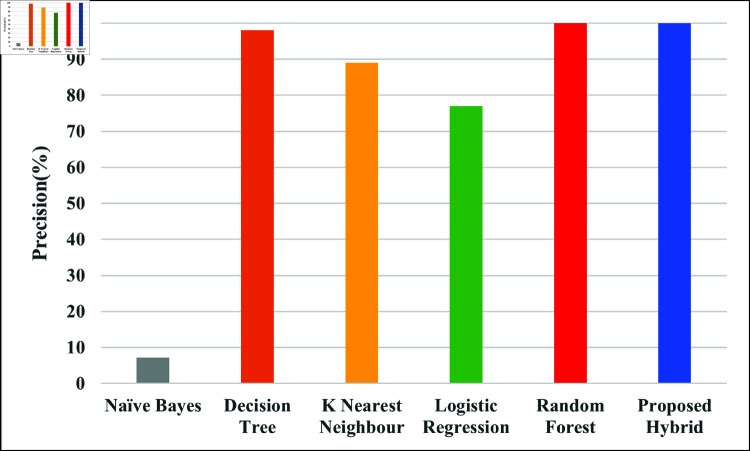
Comparison of proposed hybrid model with different ML model with regards to precision.

Fig 6 illustrate the precision rates of different ML algorithms. A better precision means fewer false positives. The precision of NB, DT, KNN, LR, RF and Proposed hybrid are 7.67%, 98%, 89%, 77%, 100% and 100% respectively.

### 4.4 Recall

Recall measures the ratio of correctly anticipated positive instances out of all actual positive instances. It is mathematically calculated as Eq 9.


$$\begin{array}{ccc} \text{Recall}=\frac{\text{TP}}{\text{TP}+\text{FN}} & & \end{array}$$ (9)

**Fig 7:**
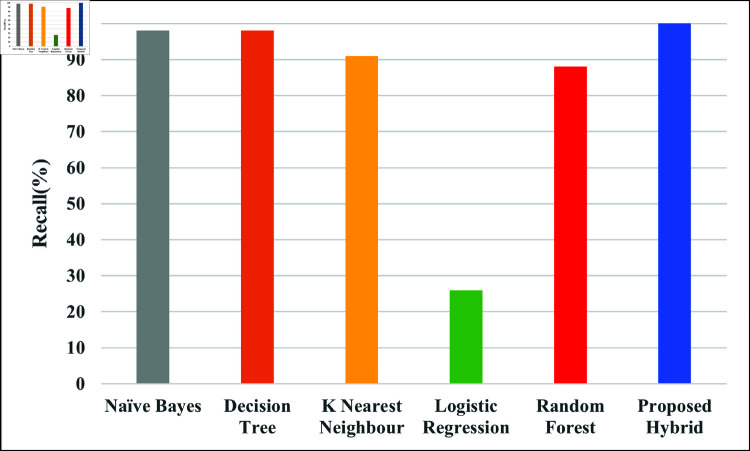
Comparison of proposed hybrid model with different ML model with regards to recall.

Fig 7 evaluates the model recall, demonstrating their ability to identify each true positive instance. High recall means fewer false negatives. The recall rates of NB, DT, KNN, LR, RF and Proposed hybrid are 98%, 98%, 91%, 26%, 88% and 99.97% respectively.

### 4.5 F1-score

The F1-score is the harmonic average of precision and recall, providing a balance between the these two. It is mathematically calculated as Eq 10.


$$\begin{array}{ccc} \text{F1-Score}=2\times \frac{\text{Precision}\cdot \text{Recall}}{\text{Precision}+\text{Recall}} & & \end{array}$$ (10)

**Fig 8:**
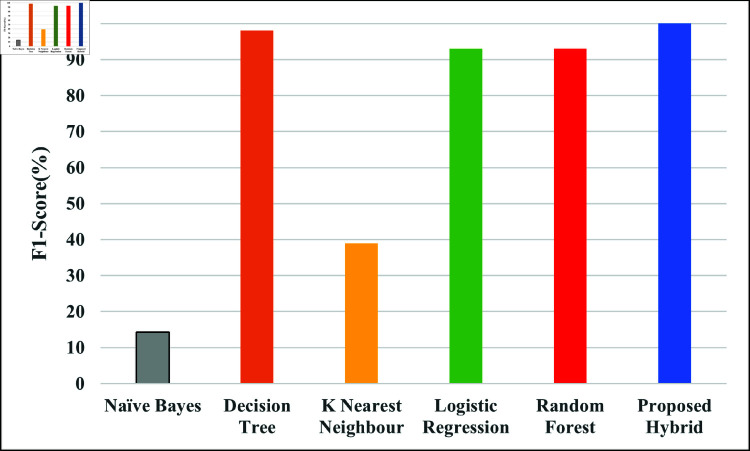
Comparison of proposed hybrid model with different ML model with regards to F1-score.

The F1-score, a measure that combines precision and recall to assess the ratio of false positives to false negatives, is displayed in Fig 8. Better overall model performance is indicated by higher F1-scores, particularly when dealing with imbalanced datasets. To demonstrate their efficacy, Fig 8 contrasts models or parameter settings. The F1-Scores attained by NB, DT, KNN, LR, RF and Proposed hybrid are 14.22%, 98%, 39%, 93%, 93% and 99.98% respectively.

Table 5 shows a comparative analysis of proposed hybrid model with state of art approach for anomaly detection. The accuracies of various ML and DL techniques from recent studies are listed alongside the proposed hybrid model from this research. The results indicate that the proposed hybrid model achieved the highest accuracy of 99.99%, surpassing other work including [24,37–40] with accuracies of 91.62%, 94%, 97%, 98.59% and 98.83% respectively. The superior performance demonstrates the efficiency of the hybrid model in precise anomaly detection and its potential to enhance the security of IoT-enabled metaverse environments. However, the table also highlights the diversity of methodologies, underlying different datasets, and focus areas, emphasizing the need for further exploration into scalable, adaptive, and generalizable solutions.

**Table 5:** Comparison of proposed hybrid model with state-of-art approach.

Ref.	Dataset	Models	Accuracy
[37]	Existing sensor device in lab	DT	91.62%
[38]	UNSW-NB15	DT	94%
[24]	NSL-KDD	XGBoost	97%
[39]	CIC-IDS-2017	NB	98.59%
[40]	UNSW-NB15	RF	98.83%
**Proposed Hybrid**	**CIC-IDS-2017**	**RF + NN**	**99.99%**

### 4.6 ROC curve

**Fig 9:**
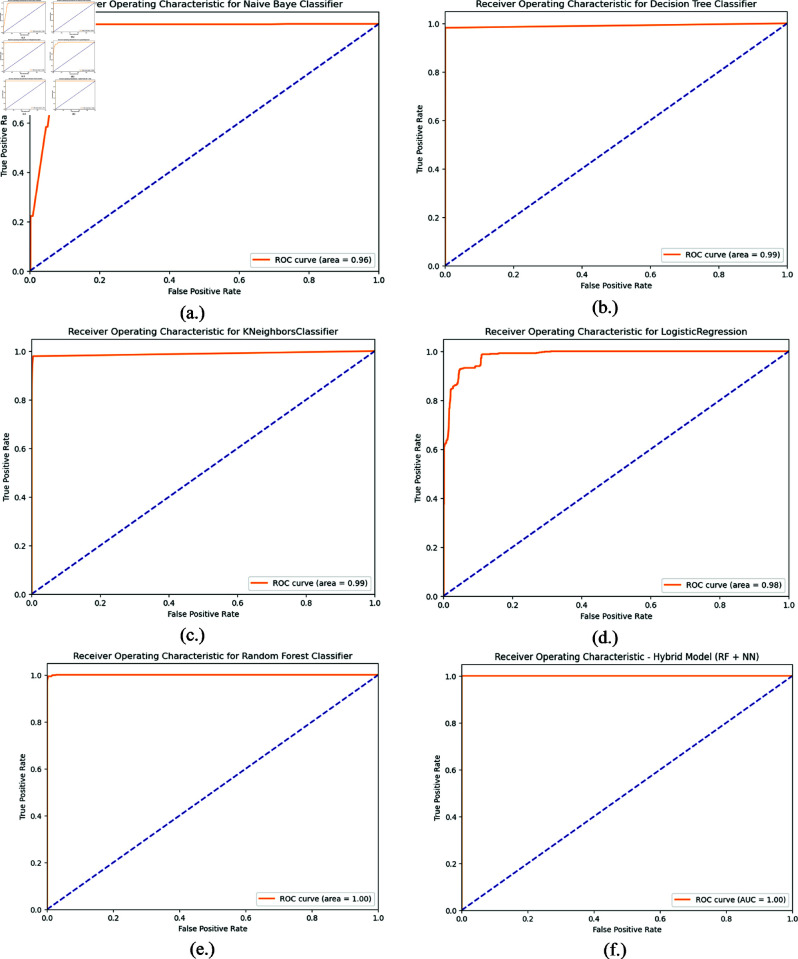
ROC curve. (a) NB, (b) DT, (c) KNN, (d) LR, (e) RF, (f) proposed hybrid.

The Receiver Operating Characteristic (ROC) curve plots the performance of a classifier across a range of threshold values visually. The ROC curve is also helpful in judging classification techniques, since it describes the trade-off between True Positive Rate (TPR) and False Positive Rate (FPR) at various thresholds. It is useful to have the possibility of comparing models and choosing optimal thresholds and monitoring overall performance. This is the case with AUC-ROC, which in itself suggests stronger discrimination if there is a better Area Under Curve (AUC) measure. Performance of models and relative error trade-off rather than countable absolute performance are resistant to class imbalances with clear visual interpretations. Fig 9 is used to draw the ROC curves of ML algorithm NB, DT, KNN, LR, RF and proposed model. Since AUC was found to be 1.0 for the case of proposed hybrid model, it concluded a higher level of classification expertise with respect to others.

### 4.7 Learning rate curve

**Fig 10:**
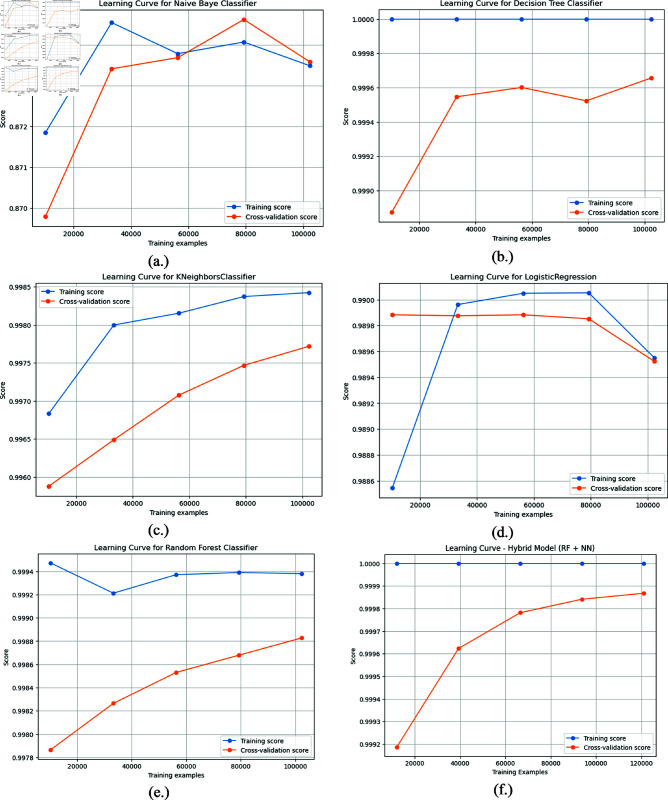
Learning rate curve. (a) NB, (b) DT, (c) KNN, (d) LR, (e) RF, (f) proposed hybrid.

Learning rate curve is generated by plotting metrics such as loss or accuracy over training iterations or epochs, often for both the training and validation datasets, visually representing a model’s training progress. It helps in assessing model convergence, identifying overfitting or underfitting, and adjusting the learning rate based on the responsiveness of the model by observing the differences between training and validation performance. It also detects problems such as low-quality data or a wrong design and gives immediate feedback towards improving the training strategy. Fig 10 shows learning rate curves of ML algorithms consisting of NB, DT, KNN, LR, RF and proposed model. From these learning curves, it can be clearly seen that how close the score is between training and cross-validation that is the minimal overfitting by proposed hybrid model against other ML models.

## 5 Application

Beyond the Metaverse, the suggested hybrid anomaly detection model (RF + NN) can be modified for a number of IoT applications, especially in the fields of smart cities and smart healthcare:

### 5.1 Smart cities

The model can be used to avoid cyber threats in intelligent traffic systems, detect anomalies in urban infrastructure, secure IoT-based smart grids, and identify intrusions in public surveillance networks. City officials can reduce cyber threats and improve municipal security by instantly spotting anomalous data trends.

### 5.2 Smart healthcare

The model is very useful for spotting fraudulent access in hospital networks and safeguarding linked medical devices. The model can assist in preventing data breaches and cyberattacks that could jeopardize patient privacy and safety by spotting questionable activity in wearable medical IoT devices and electronic health record (EHR) systems.

### 5.3 Industrial IoT

The model can be used to improve security in robotic process automation (RPA) systems, stop illegal access to industrial control networks, and identify cyberattacks in industrial settings. Industries can lessen operational disruptions brought on by cyber threats by regularly observing data patterns.

### 5.4 Smart homes

By keeping an eye on unusual device activity, unauthorized IoT device access, and possible security breaches in smart home networks, the model guarantees cybersecurity in connected home environments. By doing this, users’ confidence in home automation systems is preserved and cyber breaches are avoided.

### 5.5 Autonomous vehicles

The strategy is crucial for protecting vehicle-to-everything (V2X) communications and guarding against hacking attempts, data spoofing, and GPS signal manipulation. By maintaining the integrity of vehicle networks, the model improves passenger safety and the effectiveness of transportation.

These applications show how the suggested paradigm is useful outside of scholarly research, demonstrating its scalability, adaptability, and efficacy in safeguarding critical IoT infrastructures. The hybrid paradigm serves as a foundation for next-generation cybersecurity solutions by guaranteeing cybersecurity in various IoT scenarios.

## 6 Limitation, discussion and future scope

Security and privacy issue arises when IoT is intertwined with the metaverse, and anomaly detection methods have to be quite strong. Even though this paper shows the possibility of anomaly detection by ML models, there are still several constraints and issues that need to be addressed and rectified. Problems such as these have to be addressed if the IoT-enabled metaverse systems are to be reliable, scalable, and ethically sound. This section identifies major flaws in the study, their ramifications, and areas for further exploration to make the anomaly detection system more resilient and flexible.

1. Limited Dataset Scope and Generalization: CIC-IDS 2017 is one of the best available benchmarks to evaluate intrusion detection. However, predefined attack scenarios could not capture real-world, IoT-enabled metaverse evolving threats. Therefore, different datasets together with the application of real-time data collecting should be taken into account to improve the generality and adaptability of ML models for next research activities.
2. Scalability of the model: The proposed hybrid model in massive IoT ecosystems offers great accuracy but may turn to be computationally extensive and postpone real-time deployments. Hardware accelerators, distributed computing, lightweight methods could help to overcome this restriction toward an efficient real-time anomaly detection task.
3. False positives and model interpretability: The proposed hybrid model could still be generating FP despite such great accuracy, therefore compromising user confidence and raising running operating costs. Feature importance analysis or explainable artificial intelligence methods could improve the interpretability of the model, hence increasing user confidence and providing more thorough understanding of decision-making procedures.
4. Privacy problems and ethical consequences: Particularly in IoT-enabled metaverse environments when private user data is involved, ML models employed for anomaly detection raise privacy concerns. Future research should employ privacy-protecting techniques like differential privacy or federated learning to solve moral conundrums while keeping strong detection performance.
5. Changing the threat environment and adaptive frameworks: The stationary character of the trained ML models causes such models to be unable to adjust to new hazards in the IoT environment. Thus, the future research has to concentrate on creating adaptive learning systems like online learning or reinforcement learning that would guarantee the continual development of the models and strong resistance against new attack paths.

## 7 Conclusion

The decentralized, immersive, and networked digital ecosystems of Metaverse and IoT are poised to merge the virtual and physical worlds seamlessly. It can transform the healthcare, educational, and entertainment sectors, among others. However, interdependencies in IoT-enabled Metaverse ecosystems raise some pertinent ethical, security, and privacy concerns. It will identify unusual patterns in device behavior or network traffic and prevent potential risks related to unlawful access, data breaches, and distributed DDoS attacks. This work proposed a hybrid model combining RF and NN and also compare it with the performance of different ML models including RF, DT, KNN, NB, and LR in anomaly detection in IoT-enabled Metaverse scenarios. The CIC-IDS 2017 Network Intrusion Dataset is the most common benchmark dataset that is applied to test and train intrusion detection systems. In comparison with all other models, the proposed hybrid model proved to be the most effective and applicable, hence making it the strongest. The hybrid model had the highest accuracy in testing, which was 99.99% while in training it was 99.98%. In terms of accuracy, it performed impressively and could reduce FP, scoring a precision rate of 100%. Meanwhile, the model attained an F1-score of 99.98% and a recall score of 99.97%, the model based on the hybrid algorithm produced a great precision-recall curve balance, as this demonstrates an effective model towards achieving the identification of actual anomalies. These results indicate that the hybrid model is accurate and applicable for anomaly detection in the complex and dynamic IoT-enabled Metaverse.
